# Integration of Genomic and Other Epidemiologic Data to Investigate and Control a Cross-Institutional Outbreak of *Streptococcus pyogenes*

**DOI:** 10.3201/eid2206.142050

**Published:** 2016-06

**Authors:** Victoria J. Chalker, Alyson Smith, Ali Al-Shahib, Stella Botchway, Emily Macdonald, Roger Daniel, Sarah Phillips, Steven Platt, Michel Doumith, Rediat Tewolde, Juliana Coelho, Keith A. Jolley, Anthony Underwood, Noel D. McCarthy

**Affiliations:** Public Health England, Colindale, UK (V.J. Chalker, A. Al-Shahib, R. Daniel, S. Phillips, S. Platt, M. Doumith, R. Tewolde, J. Coelho, A. Underwood);; South East England Public Health England Centre, Chilton, UK (A. Smith, S. Botchway, E. Macdonald, N.D. McCarthy);; University of Oxford Department of Zoology, Oxford, UK (K.A. Jolley, N.D. McCarthy);; University of Warwick, Warwick Medical School, Coventry, UK (N.D. McCarthy)

**Keywords:** Group A Streptococcus, invasive group A streptococcal infection, iGAS, outbreak, genome, infection control, whole-genome sequencing, Streptococcus pyogenes, epidemiologic data, genomic data, investigation, bacteria, streptococci

## Abstract

Single-strain outbreaks of *Streptococcus pyogenes* infections are common and often go undetected. In 2013, two clusters of invasive group A *Streptococcus* (iGAS) infection were identified in independent but closely located care homes in Oxfordshire, United Kingdom. Investigation included visits to each home, chart review, staff survey, microbiologic sampling, and genome sequencing. *S. pyogenes* *emm* type 1.0, the most common circulating type nationally, was identified from all cases yielding GAS isolates. A tailored whole-genome reference population comprising epidemiologically relevant contemporaneous isolates and published isolates was assembled. Data were analyzed independently using whole-genome multilocus sequencing and single-nucleotide polymorphism analyses. Six isolates from staff and residents of the homes formed a single cluster that was separated from the reference population by both analytical approaches. No further cases occurred after mass chemoprophylaxis and enhanced infection control. Our findings demonstrate the ability of 2 independent analytical approaches to enable robust conclusions from nonstandardized whole-genome analysis to support public health practice.

## Abstract 2

Genomic surveillance can effectively detect such outbreaks, providing increased intelligence to support infection control.

The reported annual incidence of invasive group A *Streptococcus* (iGAS) infection in industrialized countries is ≈3 cases per 100,000 persons per year (*1*–*3*). Incidence is 3-fold higher among persons >70 years of age, particularly among the very elderly (*1*,*2*,*4*). Older persons have also been shown to have higher case-fatality rates, including in studies considering particular clinical syndromes, suggesting that age is a risk for death independent of the clinical form of iGAS (*1*,*5*,*6*). Population-based study data estimate incidence among long-term care facility (LTCF) residents as 3.4-fold (*7*), 6.0-fold (*8*), and 7.8-fold (*9*) higher than that among elderly persons living outside institutional settings. Annual incidence estimates among LTCF residents range from 27 (*7*) to 74 (*9*) cases per 100,000 persons. Case-fatality rates are also higher among LTCF residents (*7*–*9*).

Although iGAS mainly occurs sporadically, outbreaks have been recognized in hospitals, particularly in association with surgery and maternity care (*10*,*11*), and in LTCFs, where they appear to be increasing (*12*,*13*). Many iGAS outbreaks go unidentified. Investigations that reviewed residents’ medical records in 7 LTCFs in which 1 case of iGAS was reported identified missed outbreaks in 4 of the facilities (*4*). Furthermore, a surveillance study reported that LTCF staff members were unaware of hospital-diagnosed iGAS among residents other than through study feedback (*7*). Three surveillance studies, including 2 that used bacterial subtyping (*7*,*8*), identified iGAS clusters that had not been identified as outbreaks outside the surveillance scheme (*7*–*9*). The delayed identification of outbreaks in LTCFs (*14*,*15*) and clinical geriatric care settings (*16*,*17*) has also been described. Prospective surveillance in Ontario, Canada, identified 20 hospital outbreaks that were of smaller average size, often shorter duration, and more often outside the surgical and maternity settings than was expected based on findings in the nosocomial outbreak literature (*10*). Thus, sporadic disease may include many unidentified small outbreaks. In an LTCF surveillance study (*8*), 40 of 383 isolates were members of 18 clusters of indistinguishable strains, and in another study (*7*), 34 of 134 isolates were associated with 13 clusters, suggesting that 10%–25% of culture-confirmed cases in LTCFs may be associated with outbreaks.

Genomic data are increasingly available to support the identification and investigation of outbreaks (*18*,*19*), including 2 iGAS outbreaks associated with maternity units (*20*,*21*). In 1 of these studies, isolates collected on 2 consecutive days at a hospital were highly similar, and they were distinguishable from 2 other isolates of the same M protein gene (*emm*) type collected at a later date from 2 other hospitals (*21*). In the second study (*20*), genome sequencing confirmed the relatedness of isolates from 2 patients on a maternity ward with fatal disease and isolates subsequently obtained from another patient, household contacts, and healthcare workers; the study also discriminated these isolates from 9 epidemiologically and geographically separated isolates of identical *emm* type. Genome sequencing may, therefore, separate *Streptococcus pyogenes* isolates with close epidemiologic relationships from the background population, as suggested from findings from some other species of bacteria (*19*).

The purpose of this study was to integrate genomic data with other epidemiologic data in the investigation and control of a cross-institutional outbreak of *S. pyogenes* infection. We assessed approaches to enable robust inferences in the absence of standard analytical methods.

## Methods

We used standard epidemiologic and microbiologic approaches to investigate clusters of GAS infection in 2 managerially independent but closely located LTCFs (home A and home B) in Oxfordshire, United Kingdom, in 2013. We also applied genomic sequencing to available isolates, analyzed the data using 2 independent approaches, and performed a systematic literature review to describe evidence for the efficacy of different control strategies.

### Literature Search

On February 21, 2014, we searched PubMed, using the terms: (“pyogenes” OR “group A streptococc*”) AND (“care” OR “nursing” OR “residential”) AND (“home” OR “homes” OR “facility” OR “facilities” OR “setting” OR “settings”). One author (N.D.M.) reviewed the 131 abstracts retrieved by this search to identify those that referred to outbreaks or LTCFs (27 papers) and to population-level surveillance (3 papers). Identified papers were reviewed, references were searched for similar papers, and information was extracted on the control methods used, outcomes, and whether reported outbreaks were each due to a single strain of *S. pyogenes* or involved multiple strains.

### Epidemiologic Investigation of GAS Cases in LTCFs

We reviewed medical records for all possible cases of GAS infection among residents of the 2 LTCFs in 2013. In addition to cases already notified to public health authorities, additional possible cases of GAS were identified for review through interviews by public health staff with senior LTCF staff. Healthcare staff used case definitions from the UK national guidance (*22*) to assess residents or staff with symptoms suggestive of GAS infection. Clinically indicated samples were obtained, as were skin and soft tissue infection samples and throat swab samples from residents or staff reporting sore throats. A web-based survey of staff enabled anonymous reporting of symptoms and infection-control practices.

### Microbiology

Samples from residents and staff were cultured to detect *S. pyogenes* using standard methods. The Public Health England National Streptococcal Reference Laboratory (Bacterial Reference Department) performed *emm* gene sequence typing on each isolate obtained as previously described (*23*,*24*) using DNA prepared by using the Wizard Genomic DNA Purification Kit (Promega, Madison, Wisconsin, USA); quality was determined by using a NanoDrop 2000 Spectrophotometer (Thermo Scientific, Waltham, MA, USA), and quantity was determined by using a Qubit 3.0 Fluorometer and quantitation assays (Thermo Fisher Scientific, Waltham, MA, USA). For sequencing preparation, we used a Nextera XT DNA Library Preparation Kit (Illumina, San Diego, CA, USA), and for sequencing, we used a HiSeq 2500 System (Illumina) and the 2 × 100-bp paired-end mode. As a reference dataset to represent the background population, we used published genomes (*20*) and contemporaneous isolates of the same *emm* type from 3 clusters in other areas of the United Kingdom (Table).

**Table:** Clinical and demographic characteristics of patients with isolates sequenced in a study integrating genomic and other epidemiologic data to investigate and control a cross-institutional outbreak of Streptococcus pyogenes*

**Area, laboratory no.**	**Healthcare setting**	**Age/sex**	**Source of isolate**	**Clinical presentation**	**Outcome**
**1**					
H131441217	Care home	95 y/F	Blood culture	Bilateral periorbital cellulitis, sepsis	Died
H131520646	Care home	91 y/M	Blood culture	Facial cellulitis	Died, ANP
H131640460	Care home	65 y/M	Nasal swab sample	Rash, fever	Recovered
H131620455	Care home	84 y/F	Arm wound swab sample	Arm cellulitis	Recovered
H131720333	Care home	101 y/F	Ear swab sample	Weeping ear	Recovered
H132060515	Care home (CW)	19 y/F	Throat swab sample	Sore throat	Recovered
**2**					
H131280521	Care home	94 y/F	Blood culture	Severe soft tissue infection	Died
H131100707	Care home	84 y/F	Blood culture	Fever, leg cellulitis, diarrhea, vomiting	NR
H131220725	Care home	93 y/F	Blood culture	Fever, severe cellulitis	Died
H131620436	Care home	78 y/F	Blood culture	Emergency room admission	NR
H131020872	Maternity service	7 d/F	Umbilical wound swab sample	NR	NR
**3**					
H131180727	Hospital	87 y/M	Blood culture	NR	NR
H130500483	Hospital	60 y/M	Blood culture	Rash, sepsis, suspected CAP	NR
**4**					
H130620575	Hospital	39 y/M	Pus swab sample	NR	NR
H130620574	Hospital	71 y/M	Cannula site swab sample	NR	NR

*ANP, acute necrotizing pneumonitis diagnosed postmortem; CAP, community-acquired pneumonia; CW, care worker, NR not recorded.

### Bioinformatic Processing

We used 2 independent approaches to process 39 genome sequences, of which 6 were for isolates from the LTCFs, 9 were for isolates from contemporaneous confirmed cases, and 24 were published genomes (*20*). We used Burrows-Wheeler Aligner software (http://bio-bwa.sourceforge.net/) (*25*) to map reads to *emm* sequence type 1.0 (*emm*1) of *S. pyogenes* MGAS5505 (GenBank accession no. NC_007297-2). Single-nucleotide polymorphisms (SNPs) were discovered by using GATK2 software (Genome Analysis Toolkit v2) (*26*) and filtered by using the following parameters: genotype quality >40, ratio of consensus/nonconsensus base >0.8, distance to nearest SNP >15, root mean square of the mapping quality of the reads >50, number of reads with 0 mapping quality = 0. Reads were independently assembled by using the Velvet algorithm package (*27*), and loci were annotated with the genome annotation of *S. pyogenes* MGAS5005, which resulted in identification of 1,514 non-paralogous genetic loci with sequence data, enabling whole-genome multilocus sequence type (wgMLST) analysis (*28*).

### Bioinformatic Analysis

We constructed a maximum-likelihood phylogenetic tree by using RAxML (*29*), a multiple FASTA file of concatenated SNPs. We searched for 68 known superantigen and antimicrobial resistance genes, which were considered present if matches were found with >95% coverage and <10% SNPs difference from known variants.

We summarized allelic relationships by using the Genome Comparator tool within BIGSdb (*30*) and used pairwise differences to estimate a neighbor-joining tree by using MEGA5 software (*31*). The distribution of pairwise allelic differences among isolates was compared in each of 3 categories: within the current suspected outbreak, within an outbreak reported by Turner et al. (*20*), and comparing the current suspected outbreak isolates with all other isolates in the assembled dataset.

## Results

### Literature Review

Our literature review identified 72 LTCF-associated iGAS outbreaks from individual outbreak reports, summary data identified in surveillance studies, reviews, and laboratory studies (Technical Appendix). These outbreaks included 31 clusters, which were defined as outbreaks on the basis of shared subtypes among >2 cases occurring at a facility within 1 year. Subtyping results were available for 22 other outbreaks that were identified by other means. A single or dominant strain was identified for 19 of these outbreaks; multiple strains were reported for the other 3 outbreaks.

Interventions were varied but encompassed 3 broad approaches and showed limited evidence of different outcomes. First, treatment restricted to patients with GAS or to patients with GAS and to their direct contacts with laboratory confirmed infection was associated with disease control in some outbreaks. However, in 1 outbreak, disease recurred after 2 rounds of this selective treatment, so mass chemoprophylaxis was administered within the LTCF. Second, in 2 reported outbreaks, all staff and residents were screened for GAS and if positive, they were offered chemoprophylaxis. This screening and treatment was associated with disease recurrence and repeated screening and treatment. Additional cases of iGAS were diagnosed between decisions to screen and provide chemoprophylaxis and to actually implement chemoprophylaxis. Third, in all identified reports, chemoprophylactic treatment of all staff and residents was associated with control of iGAS, although in 1 incident, persistent infection was shown in a resident who had a gastrostomy tube.

The literature review showed that screening detected carriage rates of <10% among LTCF residents, with 2 exceptions, for which carriage rates were 20% and 16%. As previously described, carriage rates were lower among LTCF staff than residents (*12*,*13*).

### Epidemiology of Cases in Home A and Home B

**Figure 1:**
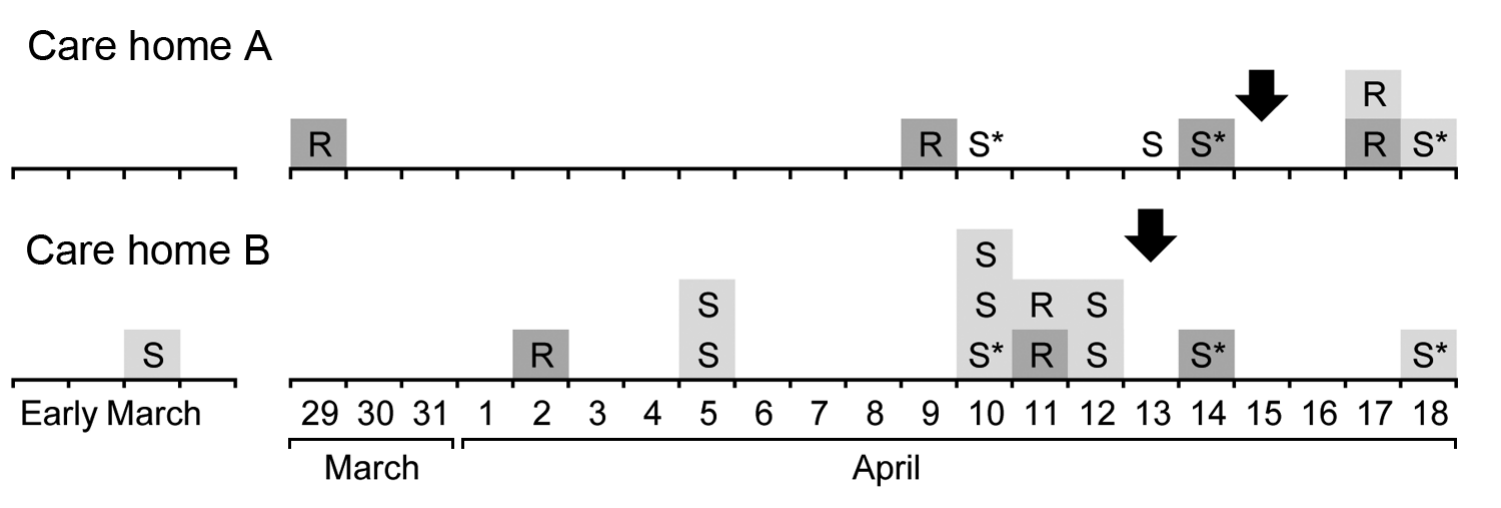
Onset dates of group A *Streptococcus* infection in 2 long-term care facilities in Oxfordshire, United Kingdom, 2013. Arrows indicate initiation of chemoprophylaxis; S, staff; R, resident; * indicates staff who worked in both homes. Dark gray shading indicates laboratory-confirmed infections; light gray shading indicates nonconfirmed infections.

After 1 iGAS case each was identified in homes A and B, advice was given to the LTCF managers by Public Health England on infection control and how to identify other GAS-compatible infections in residents and staff. Although the 2 LTCFs were geographically close to each other, neither home could initially identify any links with the other. Mass chemoprophylaxis was initiated at home A the day after a second case was reported and at home B after GAS (not iGAS) was confirmed in another resident who had cellulitis (Figure 1). Further investigation identified 3 staff members who had worked in both of the managerially independent homes; 2 of these staff members had GAS-compatible symptoms. Of 41 staff who responded to the anonymous survey, 38 identified their roles as a healthcare assistant (18 persons), cleaner (6 persons), manager or administrator (6 persons), nurse (3 persons), or other (5 persons). One respondent reported working at both homes, and 1 reported working at home B and in the community. Of the 41 respondents, 32 reported no symptoms, 7 reported sore throats, 1 reported a rash on the neck, and 1 reported illness without specifying symptoms.

Twelve possible GAS infections were not confirmed by culture. One of the infections was on the scalp of a resident at home A; the patient, who had a temperature >39°C and hallucinations, was treated with antimicrobial drugs and tested negative on subsequent samples. Another possible infection was on the eyelid of a resident at home B. Eight possible infections were in persons who worked at home A and who reported symptoms consistent with pharyngitis; 1 of the 8 staff members also worked at home B. Another person who worked at both LTCFs reported having paronychia that was treated with antimicrobial drugs, and 1 staff member at home B reported a recurrent skin infection.

### Microbiology

*S. pyogenes* isolates were obtained from 6 of 13 specimens from symptomatic residents and staff at homes A and B. Two patients in home A, 1 of whom died, had *S. pyogenes*–positive blood cultures and facial cellulitis. One patient in home B had periorbital cellulitis and GAS bacteremia. Two other patients in home B had confirmed GAS; 1 of these patients had cellulitis and systemic symptoms, including fever, but no sample from a normally sterile site, and the other patient had an outer ear infection. One staff member working in both institutions had GAS pharyngitis.

### Isolate Relatedness, Antimicrobial Resistance, and Virulence Genes

**Figure 2:**
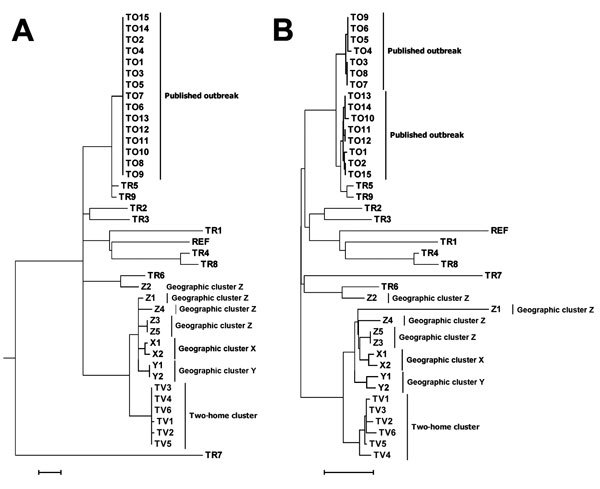
Genetic relatedness of isolates from a cross-institutional *Streptococcus pyogenes* outbreak in Oxfordshire, United Kingdom (indicated by TV plus isolate number); an outbreak described by Turner et al. (*20*) (indicated by TR and TO plus isolate number for reference and outbreak isolates, respectively); and 3 geographic outbreak clusters in the United Kingdom around the time of the TV outbreak (indicated by X, Y, or Z plus isolate number). Dendrograms are based on a single-nucleotide polymorphism maximum-likelihood phylogenetic tree constructed by using RAxML (*29*) (A) and on a neighbor-joining tree constructed from the allelic differences distance matrix (B). Scale bars indicate 10 single-nucleotide polymorphism differences (A) and 15 allelic differences (B).

**Figure 3:**
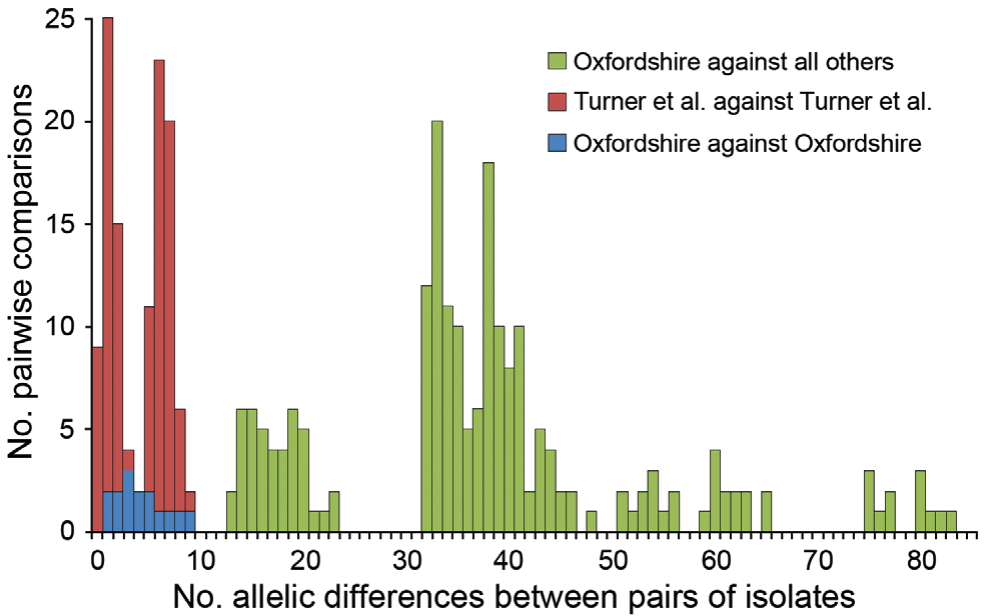
Pairwise allelic differences (across 1,514 genetic loci) among 6 isolates from a cross-institutional *Streptococcus pyogenes* outbreak in Oxfordshire, United Kingdom, and other isolates. Green indicates differences between each of the 6 Oxfordshire outbreak isolates and each of the other 33 isolates that occurred in other geographic areas in the United Kingdom around the time of the Oxfordshire outbreak or were reported by Turner et al. in 2013 (*20*). Red indicates differences between outbreak isolates from the cluster described by Turner et al. (*20*). Blue indicates differences between each isolate in the Oxfordshire outbreak compared with each of the other 5 isolates in the outbreak.

All 6 isolates from the cross-institutional *S. pyogenes* outbreak were T-type 1 (phenotypic typing), *emm*1, and *sic* (streptococcal inhibitor of complement gene) sequence type 1.02. Nine isolates from 3 contemporaneous putative clusters in the United Kingdom were available for comparison. Whole-genome sequencing yielded, on average, 85-fold depth for these 15 isolates. All shared 7-locus MLST sequence type 28 with 24 published genomes from the United Kingdom (*20*). Analysis of all 39 genomes against the *S. pyogenes* MGAS5005 genome demonstrated 334 SNPs. Separation of the isolates from the cross-institutional *S. pyogenes* outbreak from all other analyzed isolates was strongly supported (98% bootstrap value). Isolates from the staff member and 3 residents of the 2 homes were positioned in the same monophylogenetic clade and, in each case, differed from the isolates from the remaining 2 infected residents by a single SNP (Figure 2). Gene-by-gene analysis (wgMLST) also clustered the LTCF outbreak (Figure 2). The 6 isolates showed pairwise differences from each other at 1–9 (median 4) of the 1,514 loci and pairwise differences from all other isolates at >13 (median 37) loci (Figure 3). The outbreak reported by Turner et al. (*20*) showed similar within-outbreak pairwise differences at 0–9 (median 5) loci. Putative contemporaneous clusters X and Y were also supported by genome sequencing.

We determined the presence or absence of 68 virulence or antimicrobial drug–resistance–associated genes in the genomes sequenced for this study (online Technical Appendix Table). With the following exceptions, all isolates had the same genotype: *speC* was present only in isolates Y1 and Y2 (Figure 2), MF4 was present only in isolate TR7, and *spd*3 and *spy*1438 were present in all isolates except TR7. This varied presence of these 4 bacteriophage-associated genes may reflect phage mobility within *S. pyogenes* (*32*). Genes and mutations associated with macrolide and tetracycline resistance (homologs of *mefA*, *tetM,* and *tetO*) and fluoroquinolone-resistance mutations (in genes *parC* and *gyrA*) were not found in comparison with the fluoroquinolone-susceptible reference strain ATCC700294 (GenBank accession no. AF220946.1); however, a nonsynonymous SNP (T2393C) of the *par*C gene was identified in all strains. Mutations in covR/S (the *S. pyogenes* regulatory system), which are reported to be associated with strain hyperinvasiveness, were seen in all 15 isolates, in common with strain MGAS5005. No unique mutations were identified in any of the isolates.

## Discussion

We have described an iGAS outbreak across 2 managerially independent LTCFs, in which some staff worked in both facilities on separate employment contracts. Clustering in the temporal and genomic dimensions supported GAS transmission within and between these settings. Shared staff across care settings without managerial awareness may be common in this sector, in which low pay and part-time working patterns are common. Loss of pay while absent from work has been recognized as a risk factor in LTCF outbreaks (*33*,*34*). Loss of pay when excluded from work in 1 setting may also contribute to spread of infection to other settings, unless staff members understand that this exclusion applies to similar work in other settings and comply with the exclusion from work in all settings.

We found 1 other report of an iGAS outbreak across 2 LTCFs; the article explored the use of subtyping but did not include epidemiologic details of the outbreak (*35*). Spread of infection between institutions, resulting in apparently sporadic cases or small outbreaks in each, may be difficult to identify as a single outbreak. Our genomic data, triangulated with other descriptive epidemiologic data, supported our conclusion that this was a single outbreak. However, the application of these techniques is relatively untried: neither standardized methods nor extensive national, genome-sequenced reference populations are in place for *S. pyogenes*, and the population biology of this species at the whole-genome level is not fully described.

We dealt with the lack of an established analytical method, which would confirm that isolates are part of a single outbreak, and uncertain *S. pyogenes* population genomics by using 2 types of analyses (i.e., whole-genome multilocus sequence and single-nucleotide polymorphism analyses) and bioinformatics pipelines that did not rely on shared assumptions about the population biology of this species. The identification of core SNPs from a reference-based assembly, exclusion of SNPs that are nearby to avoid an excessive effect from single recombination events, and subsequent generation of an SNP-based phylogeny echo the techniques used in other analyses of this species (*20*,*21*). In our analysis of wgMLST data, we used a reference-free assembly method and assessed the number of shared and discordant alleles across the dataset without making assumptions on processes giving rise to allelic variation (*28*). The replication of clustering by different and independent approaches adds credibility to the epidemiologic inferences drawn. In the absence of extensive genome-sequenced reference populations relevant to the incident under investigation, we used a set of isolates that were identical to the isolates of interest by conventional *emm* sequence typing methods (*36*); some of the isolates from this set were also from the same time period and country as the isolates of interest. Discriminating isolates from other isolates of the same *emm* type can thus demonstrate discrimination from the population of *S. pyogenes* strains as a whole and support outbreak management when appropriate population-based, genome-sequenced reference datasets are unavailable.

Each approach clearly identified the outbreak cluster and differentiated the isolates in this outbreak from other contemporaneous isolates within the same *emm* gene sequence type (*emm*1) and MLST type, similar to findings by Turner et al. (*20*). SNP analysis indicated isolates from homes A and B were separated from all other clades by at least 14 SNPs, but they differed from each other by a maximum of 2 SNPs. Gene-by-gene analysis showed a median of 4 allelic differences in pairwise comparisons within the outbreak across 1,514 genetic loci; this finding contrasts with a median of 37 allelic differences between isolates from the current outbreak and 33 other genomes of the same *emm* type. Gene-by-gene analysis of data from the outbreak reported by Turner et al. (*20*) showed almost identical within-outbreak variation (median of 5 differences, range 0–9) (Figure 3). Thus a similar amount of allelic variation was present in the cross-institutional cluster and the cluster reported by Turner et al. (*20*). These findings contrast with much greater variation when compared with other isolates from the same *emm* type. These 2 incidents had epidemiologic data indicating likely transmission over a period of days to a few weeks. This range of variation may therefore be an estimate for expected variation in the transmission systems generating small, short-lived GAS outbreaks.

Additional well-characterized outbreaks with genome-sequenced isolates will enable fuller empirical validation of these results. More long-lived outbreaks may be associated with greater variation, and, indeed, multistrain streptococcal outbreaks can arise where a mobile genetic element can support the increase of several pathogenic lineages acquiring it (*37*). In contrast to the clear and similar discrimination of the cross-institutional outbreak isolates from other *emm*1 isolates by each analytical approach, no marked structure was shown by either analysis among the outbreak isolates. The limited extent of evolution occurring, as indexed and analyzed by our approaches across the available genome, does not enable inference on particular transmission pathways within this short-lived, single-clone outbreak.

Our literature review showed that most reported LTCF iGAS outbreaks have been caused by a single strain, and many outbreaks go unrecognized. The use of genome sequence analysis may distinguish epidemiologic clusters from background isolates, enabling detection of a large proportion of currently missed LTCF outbreaks. Given literature-based estimates that 10%–25% of apparently sporadic iGAS cases in LTCFs may belong to outbreaks (*7*,*8*), the integration of genome sequencing into iGAS surveillance in this population might be justified by this purpose alone. Detection of missed outbreaks may enable identification of risk factors that contribute to the outbreaks. Genomic surveillance could also support detection and investigation of transmission events between LTCFs and other settings. The relatively low population-level incidence of iGAS (*1*–*3*) may make genome sequence surveillance financially feasible in countries that have microbiologic iGAS surveillance systems in place.

This outbreak was controlled after use of mass chemoprophylaxis and standard infection-control measures. Infection-control measures are widely reported as part of iGAS outbreak control methods; chemoprophylaxis shows more variation. There was, on average, better control in outbreaks in which mass chemoprophylaxis was undertaken, and there were reports of further cases during the wait for screening test results (*38*,*39*). However, the evidence base is limited and carriage rates are low among residents and staff (*12*,*13*), and some argue that mass chemoprophylaxis is inappropriate (*40*). The ability to identify large numbers of outbreaks early, as appears possible by genomic surveillance, may offer a sampling frame to generate more reliable data for the role of chemoprophylaxis by analyzing outcomes across a large number of outbreaks by the approach used or through trials of different approaches.

In summary, we investigated an iGAS outbreak in 2 institutions by integrating pathogen genomic epidemiology to infer epidemiologic relatedness. Independent bioinformatic and population genetic approaches enabled credible conclusions in the absence of a standardized approach. The excess burden of iGAS among elderly residents of LTCFs, the large proportion of cases that are associated with undetected outbreaks, and the consequent opportunity to improve the evidence base for control support consideration of genomic surveillance of iGAS.

Technical Appendix Literature review results and list of genes of interest in a study integrating genomic and other epidemiologic data to investigate and control a cross-institutional outbreak of *Streptococcus pyogenes*.
