# Supplementary material for: Integration of Genomic and Other Epidemiologic Data to Investigate and Control a Cross-Institutional Outbreak of Streptococcus pyogenes
Source: Emerg Infect Dis. 2016 Jun;22(6):973–80. doi: 10.3201/eid2206.142050 (PMC4880081; doi:10.3201/eid2206.142050)
Supplement: Technical Appendix — Literature review results and list of genes of interest in a study integrating genomic and other epidemiologic data to investigate and control a cross-institutional outbreak of Streptococcus pyogenes. [file 14-2050-Techapp-s1.pdf]

Article DOI: http://dx.doi.org/10.3201/eid2206.142050

Integration of Genomic and Other Epidemiologic Data to Investigate and Control Cross-Institutional *Streptococcus pyogenes* Outbreak

Technical Appendix

Full Literature Review Results

Seventy-two iGAS outbreaks in LTCFs were identified, from individual outbreak reports (*1–14*) or summary data identified in surveillance studies or reviews (*6*,*13*,*15–20*), or laboratory studies (*16*). These 72 include 31 clusters defined as outbreaks based on shared subtypes among 2 or more cases occurring at a facility within 1 year (*15*,*18*). One of these found 13 clusters which shared sub-type, institution and time period among 134 cases, and eight instances of two isolates from the same time period and institution but with distinguishable subtypes which did not meet their subtype based outbreak definition. Among outbreaks identified by other means 22 reported subtyping. Nineteen of these identified a single or dominant strain (*1*,*3*,*5–13*,*16*), and three multiple strains (*2*,*4*,*14*).

Treatment restricted to those ill or to cases and direct contacts of cases with laboratory confirmed infection was associated with control in some outbreaks (*5*,*7*,*13*), although in one mass chemoprophylaxis was applied following recurrence after two rounds of this selective treatment (*1*). Screening all staff and residents with chemoprophylaxis if positive (*2–4*,*6*,*8*,*9*,*11–14*) was associated with recurrence and repeated screening and treatment in two reports (*2*,*8*). Further cases of iGAS were diagnosed between decisions to screen and chemoprophylax and the implementation of chemoprophylaxis (*1*,*11*). Mass chemoprophylaxis of all staff and residents was associated with control of iGAS (*1*,*3*,*10*,*13*) although in one incident persistent infection was shown in one resident with a gastrostomy tube (*1*). Screening detected carriage rates were below 10% among residents with two exceptions (20% (*5*) and 16% (*11*)) with lower rates among staff than residents as reviewed elsewhere (*6*,*13*).

References

1. Smith A, Li A, Tolomeo O, Tyrrell GJ, Jamieson F, Fisman D. Mass antibiotic treatment for group A streptococcus outbreaks in two long-term care facilities. Emerg Infect Dis. 2003;9:1260–5. PubMed http://dx.doi.org/10.3201/eid0910.030130

2. Control CfD. Invasive group A streptococcus in a skilled nursing facility—Pennsylvania, 2009–2010. MMWR Morb Mortal Wkly Rep. 2011;60:1445–9. PubMed

3. Control CfD. Nursing home outbreaks of invasive group A streptococcal infections—Illinois, Kansas, North Carolina, and Texas. MMWR Morb Mortal Wkly Rep. 1990;39:577–9. PubMed

4. Schwartz B, Elliott JA, Butler JC, Simon PA, Jameson BL, Welch GE, et al. Clusters of invasive group A streptococcal infections in family, hospital, and nursing home settings. Clin Infect Dis. 1992;15:277–84. PubMed http://dx.doi.org/10.1093/clinids/15.2.277

5. Ruben FL, Norden CW, Heisler B, Korica Y. An outbreak of Streptococcus pyogenes infections in a nursing home. Ann Intern Med. 1984;101:494–6. PubMed http://dx.doi.org/10.7326/0003-4819-101-4-494

6. Jordan HT, Richards CL Jr, Burton DC, Thigpen MC, Van Beneden CA. Group A streptococcal disease in long-term care facilities: descriptive epidemiology and potential control measures. Clin Infect Dis. 2007;45:742–52. PubMed http://dx.doi.org/10.1086/520992

7. Harkness GA, Bentley DW, Mottley M, Lee J. *Streptococcus pyogenes* outbreak in a long-term care facility. Am J Infect Control. 1992;20:142–8. PubMed http://dx.doi.org/10.1016/S0196-6553(05)80181-6

8. Greene CM, Van Beneden CA, Javadi M, Skoff TH, Beall B, Facklam R, et al. Cluster of deaths from group A streptococcus in a long-term care facility—Georgia, 2001. Am J Infect Control. 2005;33:108–13. PubMed http://dx.doi.org/10.1016/j.ajic.2004.07.009

9. Barnham M, Hunter S, Hanratty B, Kirby P, Tanna A, Efstratiou A. Invasive M-type 3 *Streptococcus pyogenes* affecting a family and a residential home. Commun Dis Public Health. 2001;4:64–7. PubMed

10. Auerbach SB, Schwartz B, Williams D, Fiorilli MG, Adimora AA, Breiman RF, et al. Outbreak of invasive group A streptococcal infections in a nursing home. Lessons on prevention and control. Arch Intern Med. 1992;152:1017–22. PubMed http://dx.doi.org/10.1001/archinte.1992.00400170099019

11. Arnold KE, Schweitzer JL, Wallace B, Salter M, Neeman R, Hlady WG, et al. Tightly clustered outbreak of group A streptococcal disease at a long-term care facility. Infect Control Hosp Epidemiol. 2006;27:1377–84. PubMed http://dx.doi.org/10.1086/508820

12. Milne LM, Lamagni T, Efstratiou A, Foley C, Gilman J, Lilley M, et al. *Streptococcus pyogenes* cluster in a care home in England April to June 2010. Euro Surveill. 2011;16:20021. PubMed

13. Cummins A, Millership S, Lamagni T, Foster K. Control measures for invasive group A streptococci (iGAS) outbreaks in care homes. J Infect. 2012;64:156–61. PubMed http://dx.doi.org/10.1016/j.jinf.2011.11.017

14. Thigpen MC, Thomas DM, Gloss D, Park SY, Khan AJ, Fogelman VL, et al. Nursing home outbreak of invasive group A streptococcal infections caused by 2 distinct strains. Infect Control Hosp Epidemiol. 2007;28:68–74. PubMed

15. Thigpen MC, Richards CL Jr, Lynfield R, Barrett NL, Harrison LH, Arnold KE, et al. Invasive group A streptococcal infection in older adults in long-term care facilities and the community, United States, 1998–2003. Emerg Infect Dis. 2007;13:1852–9. PubMed http://dx.doi.org/10.3201/eid1312.070303

16. Stanley J, Desai M, Xerry J, Tanna A, Efstratiou A, George R. High-resolution genotyping elucidates the epidemiology of group A streptococcus outbreaks. J Infect Dis. 1996;174:500–6. PubMed http://dx.doi.org/10.1093/infdis/174.3.500

17. Zurawski CA, Bardsley M, Beall B, Elliott JA, Facklam R, Schwartz B, et al. Invasive group A streptococcal disease in metropolitan Atlanta: a population-based assessment. Clin Infect Dis. 1998;27:150–7. PubMed http://dx.doi.org/10.1086/514632

18. Rainbow J, Jewell B, Danila RN, Boxrud D, Beall B, Van Beneden C, et al. Invasive group a streptococcal disease in nursing homes, Minnesota, 1995–2006. Emerg Infect Dis. 2008;14:772–7. PubMed

19. Muller MP, Low DE, Green KA, Simor AE, Loeb M, Gregson D, et al. Clinical and epidemiologic features of group a streptococcal pneumonia in Ontario, Canada. Arch Intern Med. 2003;163:467–72. PubMed http://dx.doi.org/10.1001/archinte.163.4.467

20. Davies HD, McGeer A, Schwartz B, Green K, Cann D, Simor AE, et al. Invasive group A streptococcal infections in Ontario, Canada. Ontario Group A Streptococcal Study Group. N Engl J Med. 1996;335:547–54. PubMed http://dx.doi.org/10.1056/NEJM199608223350803

Technical Appendix Table. Genes of interest, presence and absence

Gene name	Accession no. used for comparison	Protein and Function	Presence/absence in study isolates
Capsule			
HasA		Hyaluronase, production of hyaluronic acid capsule	+
HasB		Production of hyaluronic acid capsule	+
HasC		Production of hyaluronic acid capsule	+
PrtS		Cell envelope proteinase	+
Phage			
AbiR		Abortive infection phage resistance protein	+
MF4		Mitogenic factor - phage associated	– (except ERR027369 +)
Spd3		DNase (Similar to mitogenic factor), phage associated)	+ (except ERR027369 –)
Spy1438		Phage associated cell wall hydrolase	+ (except ERR027369 –)
SpyM3–1302	SpyM3_1302, gi|21909536:c1315318–1314461 *Streptococcus pyogenes* MGAS315 chromosome, complete genome	Phage protein	–
Regulatory proteins			
CcpA		Regulatory protein	+
CovR	gb|EU726239.1|:241–927 *S. pyogenes* strain H342 CovR	Two-component regulation	+
CovS	gb|EU726239.1|:933–2435 *S. pyogenes* strain H342 CovS	Two-component regulation	+
MsmR	gb|DQ984656.1|:6744–7949 *S. pyogenes* strain ALAB49 MsmR	Operon regulatory protein, msm	–
MtsR		Transcriptional regulator of metal ABC transporter MtsR-like Metalloregulator – manganese	+
Nra	gb|DQ984656.1|:126–1661 *S. pyogenes* strain ALAB49 Nra	Negative transcriptional regulator	–
PerR		Ferric uptake regulation protein	+
**Rgg**		Rgg regulator, production of SpeB Cysteine protease	+
**R**opB		Rgg regulator, production of SpeB Cysteine protease	+
Attachment			
Cpa	gb|DQ984656.1|:2092–3675 *S. pyogenes* strain ALAB49 Cpa	Fimbrial minor sturctural protein Cpa/FctA	–
FctA	gb|DQ984656.1|:4211–5158 *S. pyogenes* strain ALAB49 FctA	Fimbrial minor sturctural protein Cpa/FctA	–
FctB	gb|DQ984656.1|:6014–6583 *S. pyogenes* strain ALAB49 Nra FctB	Fimbrial minor structural protein	–
Toxins/pathogenicity factors			
ABC-transporter	gb|AF227521.1|:4607–6070	ABC-transporter	–
Emm1		M protein	+
EndoS		Endoglycoside, immunoglobulin degrading enzyme	+
Fba		Fructose-bisphosphate aldolase	+
GRAB		G protein-related a2-macroglobulin binding protein	+
IdeS		Immunoglobulin G endopeptidase, CD11b homologue	–
LaCD		Tagatose 1,6-diphosphate aldolase	+
Mac		Immunoglobulin G-endopeptidase (IdeS)	–
PrtF2	gb|DQ984656.1|:8334–10535 *S. pyogenes* strain ALAB49 PrtF2	Fibrinonectin binding protein	–
SagA		Streptolysin S precursor	+
SagB		Streptolysin S production	+
SagC		Streptolysin S production	+
Scl1		Collagen-like surface protein	–
Scl2		Collagen-like surface protein	–
ScpA		C5a peptidase	+
Sda1		Extracellular streptodornase S, DNase	+
SpeA		Exotoxin A superantigen	+
SpeB		Streptococcal pyrogenic exotoxin B, cysteine protease	+

Gene name	Accession no. used for comparison	Protein and Function	Presence/absence in study isolates
SpeC		Exotoxin C	– (except H130620574, H130620575 +)
SpeJ		Exotoxin J	+
Sic		Streptococcal inhibitor of complement	+
Ska		Streptokinase	–
Slo		Streptolysin O	+
Sof		Serum opacity factor	–
Spd		DNase	+
SpeG		Pyrogenic exotoxin	+
Spy1063		Iron(III) binding protein	+
Spy1064		Unknown	+
Spy1066		Hydrolase (HAD superfamily)	+
Spy1065		O-Acetyltranseferase (cell wall biosynthesis)	+
SpyCEP	gi|94989509:345557–351976 *S. pyogenes* MGAS10270 chromosome, complete genome	Interleukin-8 protease, cell envelope protein	–
SpyA	gi|71909814:c358588–357610 *S. pyogenes* MGAS5005 chromosome, complete genome	C3 family ADP-ribosyltransferase	+
SrtC2	gb|DQ984656.1|:5254–5979 *S. pyogenes* strain ALAB49 SrtC2	Sortase	–
UmuC-MucB	gb|AF227521.1|:6839–8128	Lesion-replicating DNA polymerases	–
Antimicrobial resistance			
FbaA		Fibrinonectin binding protein	+
GyrA		Fluoroquinalone resistance	+
Mef		Macrolide resistance	–
MefA	gb|AF227521.1|:3270–4487	Macrolide resistance macrolide-efflux protein A (mefA)	–
MefMB56Spyo029		Macrolide resistance	–
MefV1		Macrolide resistance	–
MefV2		Macrolide resistance	–
MefV3		Macrolide resistance	–
Nga		NAD glycohydrolase	+
ParC		Fluoroquinalone resistance	+
TetM		Tetracycline resistance	–
TetO		Tetracycline resistance	–
